# Artonin E Induces Apoptosis via Mitochondrial Dysregulation in SKOV-3 Ovarian Cancer Cells

**DOI:** 10.1371/journal.pone.0151466

**Published:** 2016-03-28

**Authors:** Mashitoh Abd Rahman, Faiqah Ramli, Hamed Karimian, Firouzeh Dehghan, Noraziah Nordin, Hapipah Mohd Ali, Syam Mohan, Najihah Mohd Hashim

**Affiliations:** 1 Department of Pharmacy, Faculty of Medicine, University of Malaya, Kuala Lumpur, Malaysia; 2 Department of Chemistry, Faculty of Science, University of Malaya, Kuala Lumpur, Malaysia; 3 Department of Physiology, Faculty of Medicine, University of Malaya, Kuala Lumpur, Malaysia; 4 Medical Research Center, Jazan University, Jazan, Kingdom of Saudi Arabia; 5 Center for Natural Products and Drug Discovery (CENAR), Department of Chemistry, Faculty of Science, University of Malaya, Kuala Lumpur, Malaysia; Columbia University, UNITED STATES

## Abstract

Artonin E is a prenylated flavonoid isolated from the stem bark of *Artocarpus elasticus* Reinw.(Moraceae). This study aimed to investigate the apoptotic mechanisms induced by artonin E in a metastatic human ovarian cancer cell line SKOV-3 *in vitro*. MTT assay, clonogenic assay, acridine orange and propidium iodide double staining, cell cycle and annexin V analyses were performed to explore the mode of artonin E-induced cell death at different time points. DNA laddering, activation of caspases-3, -8, and -9, multi-parametric cytotoxicity-3analysis by high-content screening, measurement of reactive oxygen species generation, and Western blot were employed to study the pathways involved in the apoptosis. MTT results showed that artonin E inhibited the growth of SKOV-3 cells, with IC_50_ values of 6.5±0.5μg/mL after 72 h treatment, and showed less toxicity toward a normal human ovarian cell lineT1074, with IC_50_ value of 32.5±0.5μg/mL. Results showed that artonin E induced apoptosis and cell cycle arrest at the S phase. This compound also promoted the activation of caspases-3, -8, and -9. Further investigation into the depletion of mitochondrial membrane potential and release of cytochrome *c* revealed that artonin E treatment induced apoptosis via regulation of the expression of pro-survival and pro-apoptotic Bcl-2 family members. The expression levels of survivin and HSP70 proteins were also down regulated in SKOV-3 cells treated with artonin E. We propose that artonin E induced an antiproliferative effect that led to S phase cell cycle arrest and apoptosis through dysregulation of mitochondrial pathways, particularly the pro- and anti-apoptosis signaling pathways.

## Introduction

Ovarian cancer is considered the most deadly gynecological malignancy [1]. Globally, more than 230,000 new cases of ovarian cancer are reported each year, with approximately 140,153 deaths annually[2]. Epidemiological studies showed that the incidence rates of ovarian cancer are highest in the western and developing industrialized countries. In 2012, almost 22,280 new cases of ovarian cancers were diagnosed in the United States, with approximately 15,520 expected deaths [3]. In Malaysia, particularly in Peninsular Malaysia, ovarian cancer is the fourth most common cancer among women, making up 5% of all female cancer cases [4].

Nearly 75% of ovarian cancer patients present with metastasis disease beyond the ovary because of the cancer’s location [5, 6]. No screening tests are currently available for early detection of ovarian cancers. Therefore, following cytoreductive surgery, chemotherapy has been the main approach of ovarian cancer treatment. Most of the current therapeutic procedures for ovarian cancer patients are based on platinum-derived drugs in conjunction with paclitaxel [7, 8]. Cisplatin and carboplatin are the most potent platinum-derived chemotherapy drugs used in treating ovarian cancer. Although chemotherapy and cytoreductive surgery are accessible to treat ovarian cancer, these approaches are considerably ineffective and highly toxic with low survival rates. In addition, the development of drug resistance that occurs over time makes the treatment of ovarian cancer more challenging. Toxicity and resistance to current chemotherapeutic drugs have encouraged researchers to explore new drug candidates from natural products, focusing on apoptosis as the physiological process that offers a powerful, non-incendiary approach to expel harmed cells from tissues, consequently securing tissue homeostasis [9]. Given that cancer cells have evolved multiple pathways to resist the induction of apoptosis, exploiting natural products that may have the capability to suppress, kill, block, and reverse the tumorigenesis process can provide novel opportunities for cancer drug development, particularly in treating ovarian cancer[10].

The genus *Artocarpus* (Moraceae) comprises nearly 55 species, which are widely distributed throughout tropical and subtropical areas, including Malaysia, Indonesia, New Guinea, and the Southern Pacific [11]. Certain species of this genus provide essential, delicious food, such as *Artocarpus chempeden* (chempedak), *A*. *heterophyllus* (jackfruit), and *A*. *altilis* (breadfruit) Many members are recognized to have medicinal value in treatment of a number of diseases, including malaria, inflammation, ulcer, and diarrhea [12, 13]. In particular, *Artocarpus elasticus* Reinw. Ex Blume is a significant source of flavorful food, timber, and traditional folk medicine for many diseases.

Artonin E is a known prenylated flavonoid. This compound is found in several *Artocarpus* plants, such as *A*. *elasticus*, *A*. *nobilis*[14], *A*. *gomezianus*[15],and *A*. *communis*[16]. Previous studies on the action of artonin E against selected cancer cell lines, including MCF-7 (breast adenocarcinoma), HCT-8 (ileocecal), MDA-MB-231 (breast adenocarcinoma), KB (human oral epidermoid carcinoma), vero cell line, and P388 (leukemia) cell lines, displayed interesting antiproliferative results[11, 17, 18]. This compound also exhibited strong radical scavenging properties against the DPPH radical [19] and proved to be a potent arachidonate 5-lipoxygenase inhibitor with an IC_50_ value of 0.36μM [20]. In addition, artonin E enhances anoikis (detachment-induced apoptosis) of H460 cells (lung cancer) in a dose-dependent manner [21]. This compound also sensitized the cells by downregulating the anti-apoptotic myeoloid leukemia cell-sequence-1 (MCL1) protein [21]. More recently, artonin E exhibited promising anti-migration and anti-invasion properties in human lung cancer cells H460 [15]. To the best of our knowledge, the mechanism of artonin E as an anticancer and apoptosis-inducing agent in human ovarian cancer cells has not been elucidated. Thus, the present study aimed to examine the apoptosis-inducing properties of artonin E in human ovarian cancer cells and the possible mechanisms involved.

## Materials and Methods

### Plant material

The stem barks of *A*. *elasticus* were collected from Ulu Langat, Selangor, Malaysia in 2010. The collection of the plant material did not require the permission of any local authority because the plant is not an endangered species. The samples were identified by Dr. Rusea Go from the Department of Biology, Faculty of Science, University Putra Malaysia. A voucher specimen (S94408)was deposited at the department herbarium [22].

### Plant extraction

The dried bark of *A*. *elasticus* (1.5 kg) was pulverized and subsequently extracted at room temperature using hexane, EtOAc, and methanol as solvents. The excessive solvents were concentrated using a rotary evaporator to yield 1.55 g, 40.22 g, and 30.52 g of dark brown semisolid extract, respectively. The EtOAc crude extract (38.22 g) was coated with silica gel and subjected to fractionation using vacuum liquid chromatography. The column was eluted with mixtures of hexane, hexane/CHCl_3,_ CHCl_3_/EtOAc, EtOAc/MeOH, and MeOH to give 60 fractions of 200 mL each. Similar fractions were combined based on the TLC profile. Crystallization of fractions 26–36 afforded 2.3 g (0.06%) of yellow powder. The compound was then recrystallized in hexane and acetone to yield artonin E with melting point (m.p.)of 232–233°C [23]and m.p.of 231–232°C, respectively. The methanol extract was fractionated using vacuum column chromatoghraphy (similar to vacuum column chromatography) to produce another batch of artonin E product (0.6 g,a yellow solid).

### Isolation of artonin E

**Fig 1:**
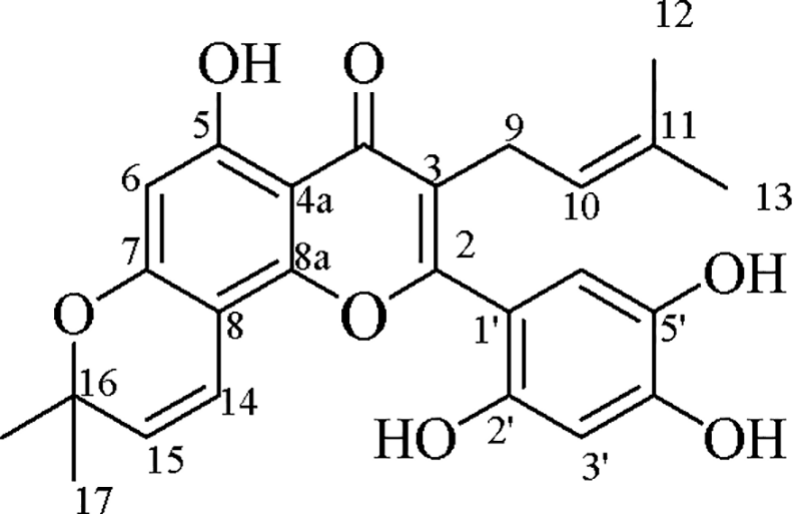
Chemical structure of artonin E.

Artonin E was isolated as a yellow powder (3 g), m.p. 232–233°C [23] m.p. 231–232°C,from methanol and the ethyl acetate bark extracts of *A*. *elasticus*. The isolates were subjected to identification and further analysis, and yielded the following results: IR *v*_max_ cm^−1^ of 3417(OH), 2979 (saturated C-H), 1644 (C = O), 1462, and 1354; UV λ_max_ nm, (log ε) of 351 (0.35), 268 (1.29), 209 (1.01); ^1^H-NMR (400 MHz, acetone*-d*_*6*_) of δ 13.19 (*s*, 1H, OH-5), 6.82 (*s*, 1H, H-6′), 6.57 (*d*, *J* = 11 Hz, 1H, H-14), 6.54(*s*, 1H, H-3’), 5.62 (*d*, *J* = 10.1 Hz, 1H, H-15), 5.08 (*t*, *J* = 6.3 Hz, 2H, H-10), 3.1 (*d*, *J* = 7.36Hz, 2H, H-9), 1.52 (*s*, 3H, H-13), 1.41 (*s*, 3H, H-12), 1.39 (*s*, 3H, H-17), and 1.39 (*s*, 3H, H-18); APT-NMR (100 MHz,acetone*-d*_*6*_) of δ 182.4 (C = O), 161.8 (C-2), 161.4 (C-7), 159.1 (C-5), 152.4 (C-8a), 148.9 (C-2′), 148.6 (C-4′), 138.2 (C-5′), 131.5 (C-11), 127.2 (C-15), 121.6 (C-10), 120.8 (C-3), 116.2 (C-6′), 114.6 (C-14), 110.5 (C-1′), 104.7 (C-4a), 103.8 (C-3′), 100.7 (C-6), 98.8 (C-8), 77.9 (C-16), 29.1 (C-18), 29.1 (C-17), 27.4 (C-13), 23.7 (C-9), and 16.8 (C-12); EIMS *m/z*(% intensity) of 436 (M^+^, 45), 421 (100), 393 (24), 203 (66), 182 (22), and 69 (15). Based on aforementioned physical and spectral data, the compound was identified as a known flavone named artonin E (Fig 1) [23].

### Cell culture

Metastatic ovarian adenocarcinoma (SKOV-3) and normal Chinese hamster ovary (CHO) cell lines were originally obtained from the American Type Culture Collection (ATCC; Manassas, VA). SKOV-3 cells were cultured in McCoy’s 5A medium and CHO cells were cultured in F12K medium. A human periodontal ligament fibroblast cell line was purchased from Lonza, USA and maintained in stroma cell basal medium [24]. T1074 (normal immortalized human ovarian surface epithelial cell line) was originally purchased from Applied Biological Materials (abm^®^) Canada and cultured in Prigrow 1 medium[25, 26].

### Cell viability assay

The inhibitory effects of artonin E and two positive controls paclitaxel and carboplatin were examined by MTT assay. Cells at a density of 5×10^3^cells/well were placed in a 96-well plate and left for 24 h to attach. The attached cells were exposed to artonin E, paclitaxel, and carboplatin at different concentrations and incubated for 24, 48, and 72 h. Subsequently, 20 μL of 5 mg/mL MTT solution was added after drug treatment and the resulting mixture was incubated for 4 h to form purple formazan. Afterward, 100 μL of dimethyl sulfoxide (DMSO) was transferred into each well to dissolve the purple formazan, and results were measured using a microplate reader (Tecan Infinite M 200 PRO, Männedorf, Switzerland) at an absorbance of 570 nm. The half-maximal concentrations that caused cell growth inhibition (IC_50_ values) were obtained from the MTT viability growth curve.

### Morphological study using a normal inverted microscope

The SKOV-3 cells were placed in a 6-well plate and left for 24 h to attach. The IC_50_ of artonin E at 48 h post treatment (8 μg/mL) based on the results of the MTT cell viability assay was used to treat the cells throughout the experiments, except for the clonogenic assay. The attached cells were treated with artonin E for 24, 48, and 72 h and observed under a normal inverted microscope at 200× magnification. The cellular morphological changes were assessed and compared with the untreated viable cells.

### Clonogenic assay

The SKOV-3 cells were trypsinized, centrifuged, and supplemented with fresh media. The cells were immediately counted and seeded (300 cells/plate) in a 60 mm Petridish, and left overnight to attach. The attached cells were treated with 0, 1, 5, 10, 15, and 20 μg/mL of artonin E for 24 h. Following incubation, the treated media were removed and fresh media were added. The cells were incubated for three weeks to form colonies. Surviving colonies were fixed with 90% ethanol and stained with crystal violet.

### Morphological assessment of apoptotic cells by acridine orange and propidium iodide double staining

The effect of artonin E in inducing cell death in SKOV-3 cells was determined using acridine orange (AO) and propidium iodide (PI) double staining. SKOV-3 cells were plated at 5×10^5^cells in a T75cm^2^ flask and incubated overnight to allow attachment. The cells were then exposed to 8 μg/mL of artonin E for 24, 48, and 72 h. After incubation, the treated cells were washed with PBS, and then trypsinized and centrifuged at 1,500 rpm for 5 min. The cells were then resuspended in cold PBS and centrifuged twice to obtain fresh clean pellets. The fresh pellets were mixed with an equal volume of fluorescent dye staining solution (1:1), comprising 10μg/mL AO and 10 μg/mL PI (dissolved in PBS). The freshly stained cell suspension was dropped onto a glass slide and observed under a UV–fluorescence microscope (Leica attached with Q-Flora Software) within 30 min, prior to fading of the fluorescence. The morphological criteria used for the classification of healthy, apoptotic, and necrotic cells are as follows: (i) viable cells show a green nucleus with round intact structure; (ii) early apoptosis displays a dense bright-green nucleus with chromatin condensation; (iii) late apoptosis exhibits a dense orange area due to condensation of chromatin; and (iv) secondary necrosis shows an orange/red intact nucleus[27].

### Annexin V assay

An Annexin V assay was performed using a BD Pharmingen^TM^ Annexin V-FITC Apoptosis Detection Kit (ApoAlert Annexin V, Clontech, California, USA). The cells were placed in a 6-well plate and left overnight to attach. Cells were then treated with 8 μg/mL of artonin E for 24, 48, and 72h. The treated cells were trypsinized and centrifuged at 1,500 rpm for 10 min to remove residual media. Subsequently, 1X binding buffer was added to rinse the fresh pellets before their resuspension in 200 μL of binding buffer. The cells were then mixed with 5 μL of Annexin V and 10 μL of PI and incubated for 15 min in the dark at room temperature. The prepared cells were immediately analyzed using flow cytometric analysis (FACS Canto 11 Becton-Dickson). Then, 300 μL of binding buffer was added to each sample to adjust the reaction volume to at least 500 μL for the flow cytometric analysis. The cells treated with DMSO (0.1%, v/v) were used as control.

### Cell cycle analysis

SKOV-3 cells at a concentration of 5×10^5^ cells were plated into a T75cm^2^ flask and incubated overnight to allow attachment. The attached cells were treated with 8 μg/mL of artonin E at different time points. After treatment, the cells were trypsinized and centrifuged at 1,500 rpm for 10 min. The fresh pellets were washed twice with PBS, fixed with 500 μL of 70% cold ethanol, and incubated at −20°C overnight. The remaining ethanol was then removed and the cells were resuspended in PBS containing 20 μL of RNase A (10 μg/mL) and 2 μL of PI (2.5 μg/mL). The resulting mixture was incubated for 30 min at 37°C in the dark. Samples were immediately analyzed using FACS Canto 11 Becton-Dickinson flow cytometry. For each sample, 10,000 cells were measured. ModFit LT software (Verity Software House, Topsham, ME) was used to analyze the percentage of cells at different phases.

### Detection of reactive oxygen species generation

2′,7′-Dichlorofluorescin diacetate (DCFH-DA) was used to examine the production of intracellular reactive oxygen species in SKOV-3 cells treated with artonin E. The SKOV-3 cells were seeded in the 96-well black plate and incubated overnight to attach. The cells were then treated with 8 μg/mL of artonin E for 24, 48, and 72 h. Hank’s balanced salt solution without serum was used to wash the cells. Then, 100 μL of DCFH-DA solution was added to each designated well, and the plate was incubated at 37°C for 30 min. The results were analyzed using a fluorescence microplate reader (Tecan Infinite M 200 PRO, Männedorf, Switzerland) at 485 nm excitation and 520 nm emission.

### Multiparametric Cytotoxicity 3 high-content screening

Cellomics Multiparameter Cytotoxicity 3 kit (Thermo Scientific^TM^, Pittsburgh, PA, USA) was used to perform simultaneous detection of the crucial apoptotic events in SKOV-3 cells treated with artonin E. Briefly, SKOV-3 cells were seeded at a density of 5000 cells/well in a black flat-bottomed 96-well plates (PerkinElmer, Inc., Wellsley, MA, USA) and left overnight to attach. The attached cells were treated with 8 μg/mL artonin E for 24, 48, and 72 h. The treated cells were then exposed to 50 μL of live cell staining solution for 30 min. The excess medium and live cell staining solution were gently removed and the cells were then fixed with 16% paraformaldehyde. After fixation, the cells were exposed with permeabilization buffer followed by blocking buffer for 15 min at room temperature. Primary Cytochrome *C* antibodies and secondary DyLight 649 conjugated goat antimouse IgG were added and allowed to interact for 1 h each. Hoechst 33342 dye was used to stain nuclei in SKOV-3 cells. Stained SKOV-3 cells in the 96-well plates were analyzed using ArrayScan high-content screening (HCS) system. All experiments were conducted in triplicates and experiments were repeated twice.

### Cytochrome c releasing apoptosis assay

Cytochrome c releasing apoptosis assay kit (ab65311) was purchased from abcam^®^ (Cambridge, UK). The assay was conducted according to the manufacturer’s instruction. Briefly, SKOV-3 cells were induced with artonin E at different time points. The induced and un-induced cells were collected and centrifuged at 6,00×*g* for 5 minutes at 4°C. The cells were washed twice with 10 mL of ice-cold PBS, centrifuged and collected the pellet. The fresh pellets were then re-suspended with 1.0 mL of 1X cytosol extraction buffer mix containing DTT and Protease Inhibitor and incubated on ice for 10 minutes. Cells were then Dounce-homogenized before centrifuging at 7,000×*g* for 10 min at 4°C. The supernatant were collected and saved as cytosolic fractions. The remaining pellets were resuspended in 0.1 mL mitochondrial extraction buffer, vortexed for 10 seconds and saved as mitochondrial fractions. Both cytosolic and mitochondrial fractions were applied for protein analysis and proceeded with standard Western blot procedure.

### Activation of caspases-3, -8, and -9 assay

Calorimetric assay of the activation of caspases-3, -8, and -9 was conducted using R & D system kit, USA. Briefly, in a T75cm^2^ flask 90% confluent SKOV-3 cells were induced with artonin E for 24, 48, and 72 h. The induced cells were trypsinized and centrifuged at 1,800 rpm for 10 min. The fresh pellets were washed with cold PBS and immediately lysed with protein lysis buffer. The cell lysate was then kept on ice for 10 min, centrifuged at 10,000 g for 15 min, and transferred into a new tube. The fresh cell lysate (50 μL), which contained 100–200 μg of protein, was added into a 96-well plate in triplicate. Fifty microliters of reaction buffer and 5 μL of caspase were then transferred into each designated well before incubating the plate in a CO_2_ incubator at 37°C for 2 h. A microplate reader (Tecan Infinite M 200 PRO, Männedorf, Switzerland) was used for analysis at a wavelength of 405 nm.

### DNA fragmentation assay

SKOV-3 cells were treated with artonin E at different time points, and harvested by adding trypsin to promote detachment. The detached cells were spun down and washed two times with cold PBS. The DNA from cells in the suspension was extracted using the suicide Track^TM^ DNA ladder isolation kit (Calbiochem, EMD Bioscience, Germany). The cells were then lysed by mixing 55 μL of TE (Tris and EDTA) lysis buffer and 20 μL of enzyme A (RNase A). The resulting mixture was incubated at 37°C for 1 h. Subsequently, 25 μL of enzyme B (proteinase K) was added and the lysate was kept in a water bath at 50°C overnight. Following incubation, 2 μL of Pellet Paint, 60 μL of 3M sodium acetate, and 662 μL of 2-propanol were added to the lysate, and the solution was briefly mixed by inversion and incubated at room temperature for 2 min. The DNA was then precipitated by centrifugation of the samples at 16,000×*g* for 5 min. The supernatant was removed and the fresh pellets were rinsed two times with 70% and 100% ice-cold ethanol. The pellets were air dried at room temperature for a few minutes to discard the ethanol and resuspended with 50 μL of DNA resuspension buffer. The prepared DNA ladder samples were loaded onto a 1.5% gel electrophoresis set up and run at 50 constant volts for 3 h. Once electrophoresis was completed, the gel was immediately stained with SybrGreen™ and the band obtained was visualized using the UV Gel Documentation system (Biospectrum 410, UVP).

### Western blot

SKOV-3 cells were plated in a 75cm^2^ culture flask and exposed to artonin E for 24, 48, and 72 h. Lysis buffer (50 mM Tris–HCl, pH 8.0; 120 mM NaCl, 0.5% NP-40; and 1 mM PMSF) was used to extract the total protein. Protein concentration was determined using a BCA protein assay reagent kit (Bio-Rad, USA). Equal amounts of protein (40 μg) extract were subjected to SDS-PAGE and electroblotted onto a polyvinylidenedifluoride membrane (Bio-Rad). Blots were then blocked with 5% non-fat milk in TBS-Tween buffer 7 (0.12 M Tris-base, 1.5 M NaCl, and 0.1% Tween 20) for 1 h at room temperature. After incubating with the appropriate primary antibody overnight at 4°C, the blots were washed and then incubated with horseradish peroxidase conjugated secondary antibody for 1 h at room temperature. The protein bands were detected using pico or femto chemiluminescence (ECL system) and visualized using a UV gel documentation system. The subsequent primary antibodies, which include those for β-actin (1:10,000), Bax (1:10,000), Bcl-2 (1:10,000), HSP-70 (1:10,000), survivin (1:10,000),caspase -3 (1:10,000), caspase -8 (1:10,000), caspase -9 (1;10,000) and cytochrome c (1:200) were purchased from Abcam, Cambridge, United Kingdom.

### Statistical analysis

Mean datafrom at least three measurements for each sample tested were normalized to the untreated results. Statistical analysis was performed using SPSS-16.0 package and GraphPad prism 5.0. Data were presented as mean±SD and *p<0*.*05*was considered significant.

## Results

### Artonin E selectively inhibits the growth of cancer cells and normal cells *in vitro*

The antiproliferative effects of artonin E on various cell lines were evaluated using the MTT assay, as shown in Table 1. This experiment was established based on the capability of the NADP(H)-dependent cellular oxidoreductase enzyme to reduce the yellow tetrazolium dye to its insoluble purple formazan, which reflects the proportion of viable cells present. Among the cell lines tested, the lowest IC_50_ values were observed for SKOV-3 cells after 24 h treatment. The IC_50_ values of SKOV-3 cells treated with artonin E markedly decreased after 48 and 72 h treatment, respectively, as shown in Table 2. By contrast, normal human ovarian cells (T1074), normal human periodontal ligament fibroblast cells, and normal CHO cells treated with artonin E were less toxic. The IC_50_ values of SKOV-3 cells treated with artonin E were slightly lower than that treated with carboplatin, which is a well-known chemotherapeutic drug (Table 2). Paclitaxel and carboplatin were used as positive control. Both of these drugs decreased cell viability in SKOV-3 cells in a time-dependent manner (Table 2).

**Table 1:** MTT assay of artonin E on various cell lines.

Cell lines	Origin of cells	IC_50_ values (μg/mL)
**SKOV-3**	Human ovary adenocarcinoma cells	12.83±0.28
**T1074**	Immortalized normal human ovarian surface epithelial cells	44.8±0.76
**Human periodontal fibroblast**	Human periodontal ligament fibroblasts	67.0±1.0
**CHO**	Normal CHO cells	57.6±2.0

The antiproliferative effects of artonin E on different cell lines *in vitro* at 24 h treatment. IC_50_ values were obtained from MTT assay. Data are reported as means±SD for measurements in triplicate.

**Table 2:** IC_50_ values of SKOV-3 cells and T1074 cells treated with artonin E, paclitaxel, and carboplatin at different time points.

Cell lines	Compound/drugs	24 h	48 h	72 h
	Artonin E	12.83±0.28	8.8±0.29	6.5±0.5
**SKOV-3**	Paclitaxel	4.6±0.28	2.5±0.2	0.8±0.2
	Carboplatin	15.1±0.36	10.5±0.5	7.2±0.25
	Artonin E	44.8±0.76	37.5±0.53	32.5±0.5
**T1074**	Paclitaxel	>50	49.3±0.76	30.5±0.5
	Carboplatin	45.6±0.57	25.8±0.28	10.8±0.7

IC_50_ values (μg/mL) were obtained from MTT assay. Data are reported as means±SD for measurements in triplicate.

### Artonin E decreases cell survival

**Fig 2:**
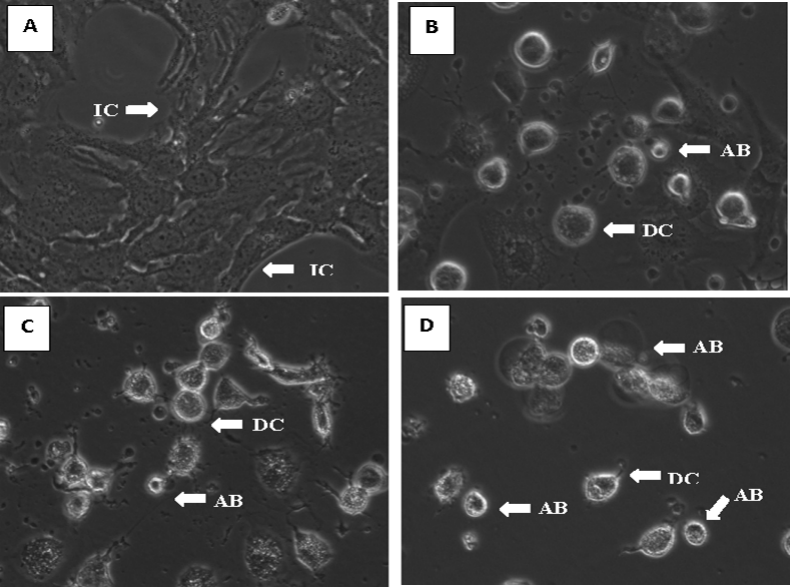
Microscopic evaluation of SKOV-3 cells treated with artonin E at different time points (200× magnification). (A) Untreated cells. (B) Cells after 24 h treatment. (C) Cells after 48 h treatment. (D) Cells after 72 h treatment. IC: Intact cell structure; DC: dissociate cell structure; MB: membrane blebbing; and AB: apoptotic body.

The morphological changes in SKOV-3 cells treated with artonin E were observed under normal inverted microscopy (Fig 2). The dissociated morphological structure of the treated cells was apparent after 24 h exposure to artonin E. Cell membrane blebs were observed with a sharp decrease in cell numbers, indicating that growth inhibition had occurred. The formation of apoptotic bodies was observed after a longer exposure time. By contrast, the normal SKOV-3 cells remained healthy with an intact structure.

### Artonin E inhibits colony formation in SKOV-3 cells

**Fig 3:**
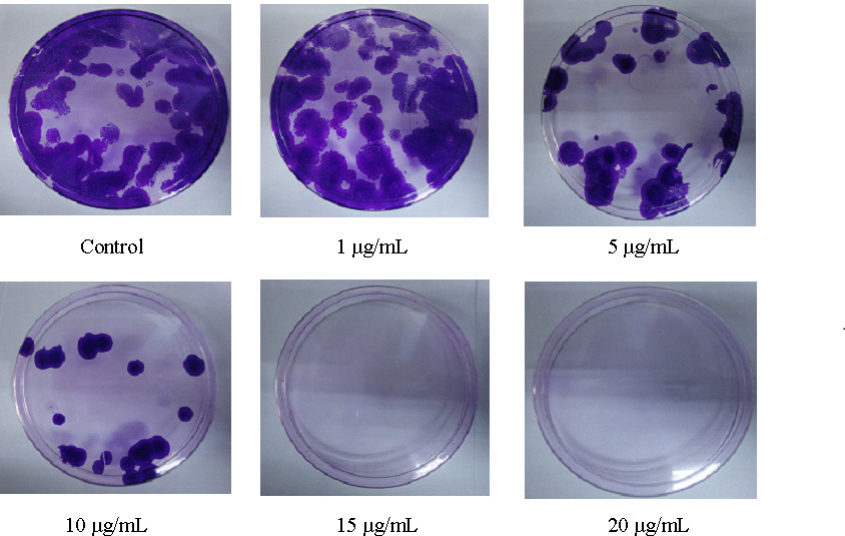
Dose-dependent evaluation of SKOV-3 cells treated with artonin E as measured by clonogenic assay. Cells were exposed to different concentrations of artonin E for 24 h and then incubated for three weeks to form colonies. The colonies formed were fixed with ethanol and stained with crystal violet.

Clonogenic assay was performed to examine the long term effect of SKOV-3 cells treated with artonin E. The results in Fig 3 show that artonin E inhibits colony formation in a dose-dependent manner. At 5 μg/mL of artonin E, almost half of the colonies were reduced, decreasing sharply at a concentration of 10 μg/mL. No colonies were formed after the cells had been treated with 15 and 20 μg/mL of artonin E, thus suggesting that this compound has an anti-proliferative effects in SKOV-3 cells.

### Quantification of apoptosis by using AO–PI double staining

**Fig 4:**
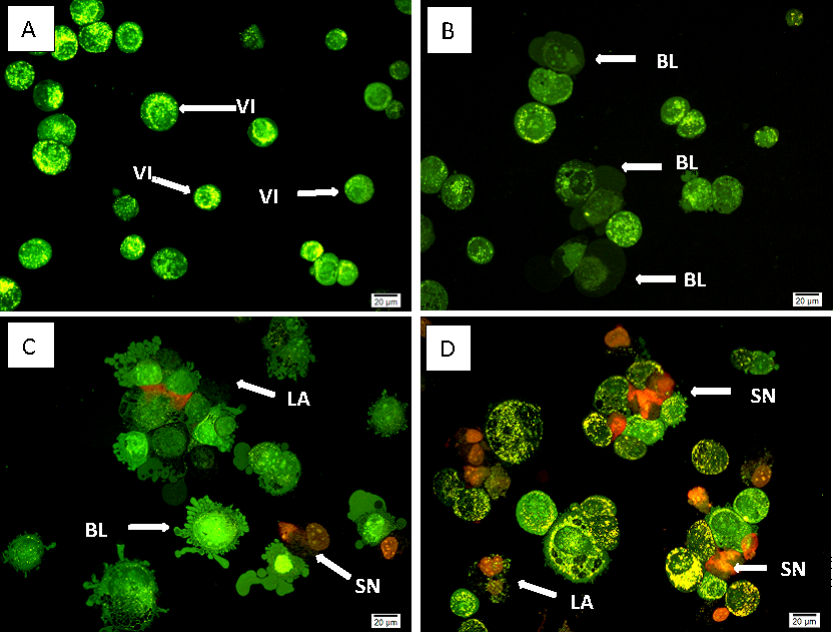
Morphological analysis of SKOV-3 cells double stained with AO and PI as observed under a flourescent microscope. The treated cells were exposed to 8 μg/mL of artonin E for 24, 48, and 72 h. (A) Untreated cells. (B) Cells after 24 h treatment. (C) Cells after 48 h treatment. (D) Cells after 72 h treatment. VI: viable cells; BL: blebbing of the cell membranes; LA: lateapoptosis; and SN: secondary necrosis.

**Fig 5:**
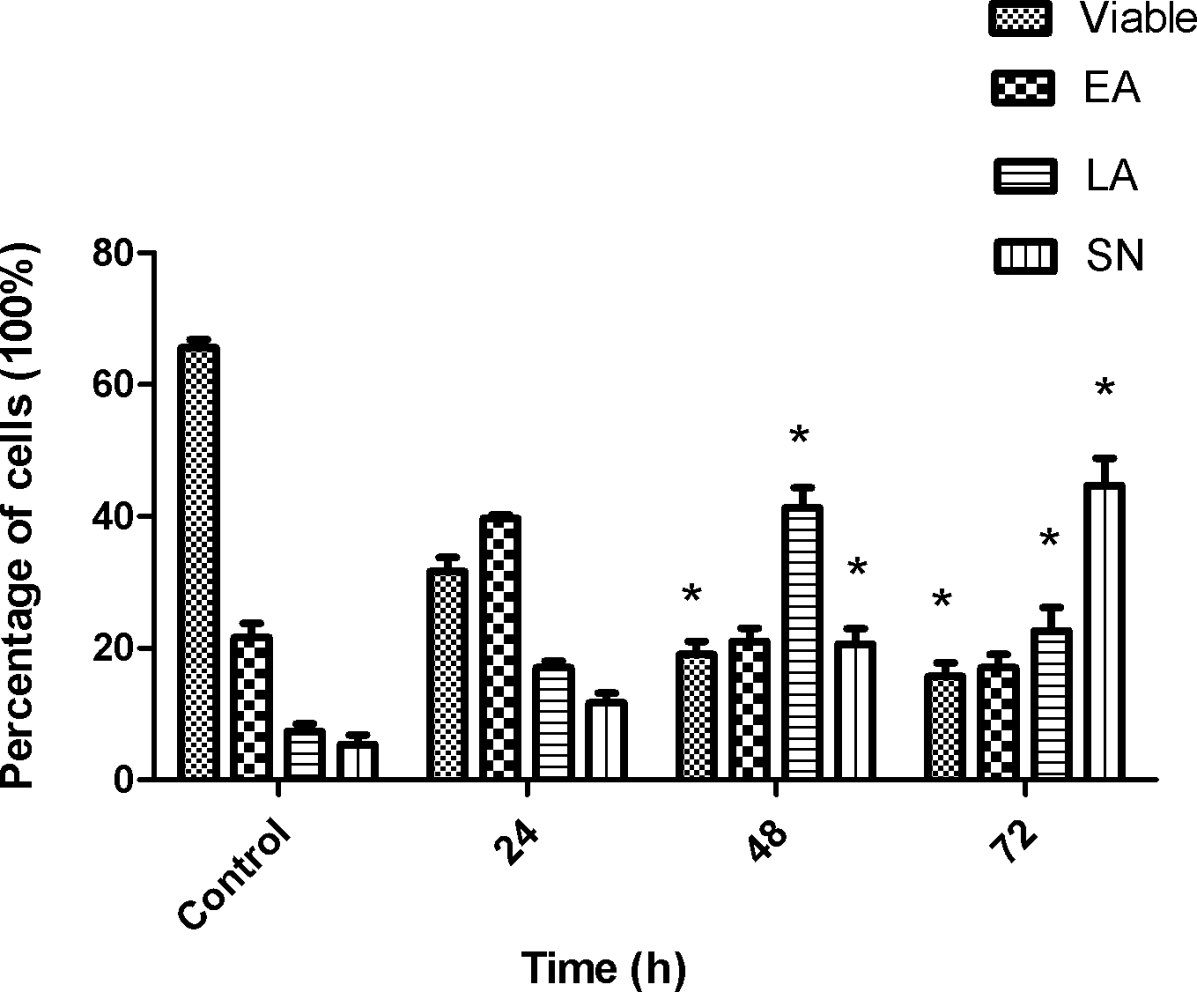
Quantitative analysis of double stained (fluorescence dyes AO and PI) untreated and treated SKOV-3 cells with artonin E. (EA: early apoptosis; LA: late apoptosis; and SN: secondary necrosis). Results are presented as mean±SD of three replicates. * indicates significant difference from the control of each phase (*p<*0.05).

AO–PI analysis was employed to examine the changes in nuclear morphology in SKOV-3 treated cells. The apoptotic cells were evaluated based on nuclear condensation and fragmentation. In this study, 200 cells from each experiment were scored and quantified randomly. Results reveal (Fig 4) that artonin E triggered morphological changes that relate to apoptosis as early as 24 h after treatment. The hallmark of early apoptosis was observed with AO intercalated within the fragmented DNA. At this time point, membrane blebbing and margination of the nucleus were clearly seen. Further 48 h of exposure showed that the treated cells had undergone late apoptosis with the observed blebbing and red/orange color. Secondary necrosis with characteristic bright red color was observed 72 h after treatment because of PI binding to the DNA of the dead cells. By contrast, the untreated cells exhibited a green intact nuclear structure. A statistically significant *(p<0*.*05)* difference in the induction of apoptosis in the treated cells (Fig 5) was observed. In addition, a concurrent increase in cell death (secondary necrosis) was observed *(p<0*.*05)* after prolonged exposure time, that is, 72 h after treatment (Fig 5). These results exhibited time-dependent typical generated morphological features associated with apoptosis upon artonin E treatment in SKOV-3 cells.

### Artonin E induces apoptosis in SKOV-3 cells

**Fig 6:**
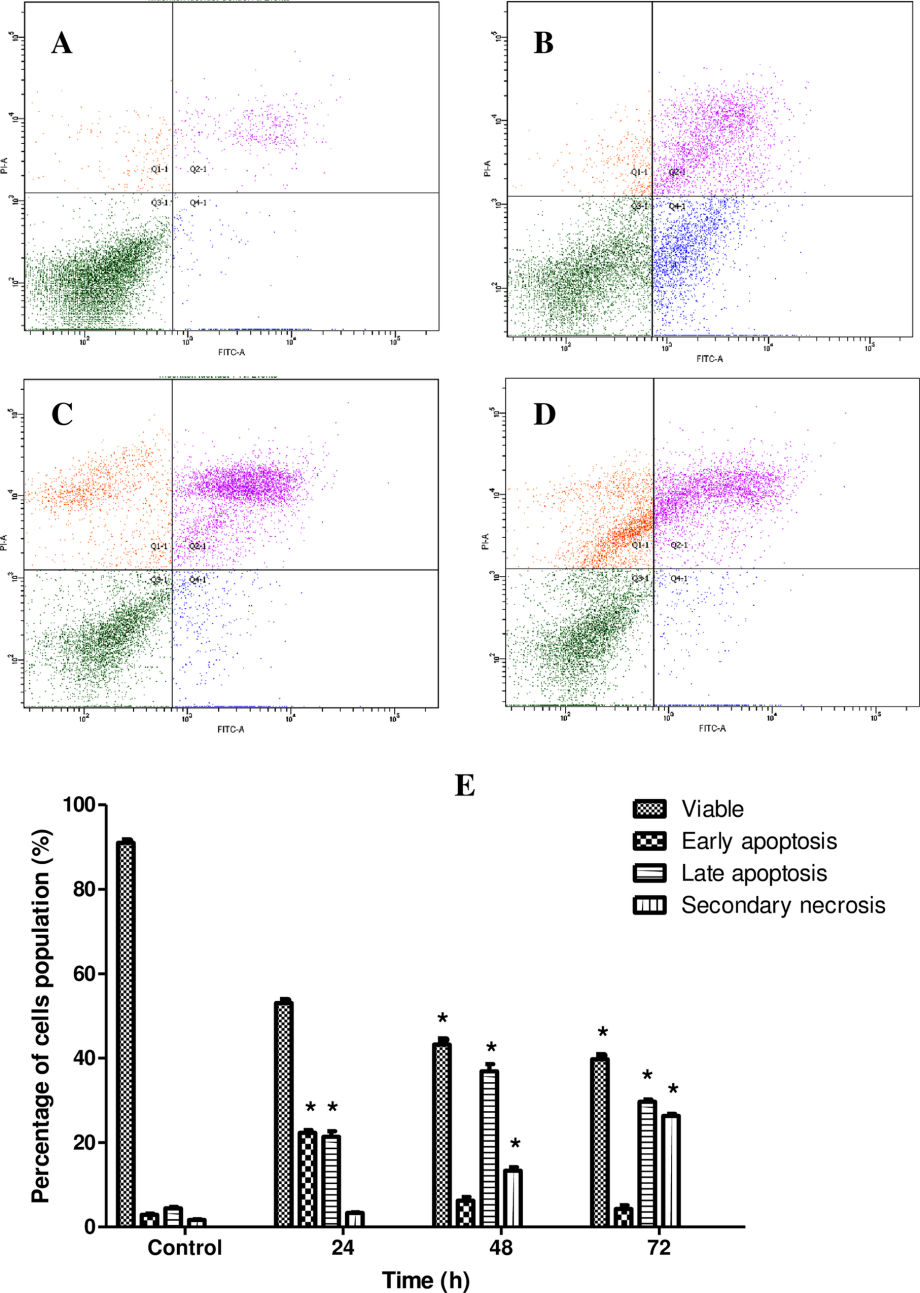
Flow cytometry analysis of Annexin V-FITC in SKOV-3 cells treated with artonin E (8 μg/mL) in a time-dependent manner. [A] Control (untreated), [B] 24 h treatment, [C] 48 h treatment, and [D] 72 h treatment. [E] Histogram. Results are presented as mean±SD of three replicates. *p<0.05 indicates significant difference from control.

Apoptosis has two distinct morphological and biochemical characterizations. Changes in morphological apoptotic cells such as loss of plasma membrane asymmetry and attachment, plasma membrane blebbing, condensation of the cytoplasm and nucleus, are always accompanied by several biochemical modifications[28]. Our results thus far demonstrated the typical morphological features of apoptosis in artonin E treated SKOV-3 cells. Double staining Annexin V/PI flow cytometry was employed in order to investigate the biochemical changes in artonin treated SKOV-3 cells. One of earlier biochemical changes in apoptosis events is a different kinetics of phosphatidylserine (PS) exposure on the outer leaflet of the plasma membrane. Annexin V, a Ca^2+^-dependent phospholipid-binding protein was known to interact specifically and strongly with PS and can be utilized to detect apoptosis by targeting the loss of plasma membrane asymmetry[29]. The AV-/PI- staining indicates viable cells due to PI does not permeable into intact cell membrane, whereas AV+/PI- staining represents the early apoptotic cells, due to loss of plasma membrane asymmetry and strong affinity of AV-FITC with PS. Instead, the AV+/PI+ represents late apoptotic and AV-/PI+ represents necrotic stage which is due to decrease of plasma membrane and nuclear membrane integrity which allow PI to pass through the membranes and intercalate into nucleic acid. As illustrated in Fig 6, more than 40% of cells at the early and late stages of apoptosis after 24 and 48 h post treatment, respectively, indicating time-dependent significant *(p<0*.*05)* increment when compared with untreated cells. In addition, treatment with artonin E also showed time-dependent decrease in viable cells with concurrent increase in necrotic cells. Therefore, current results suggest that antiproliferation and apoptosis in SKOV-3 treated cells are closely related.

### Artonin E induces S phase cell cycle arrest in SKOV-3 cells

**Fig 7:**
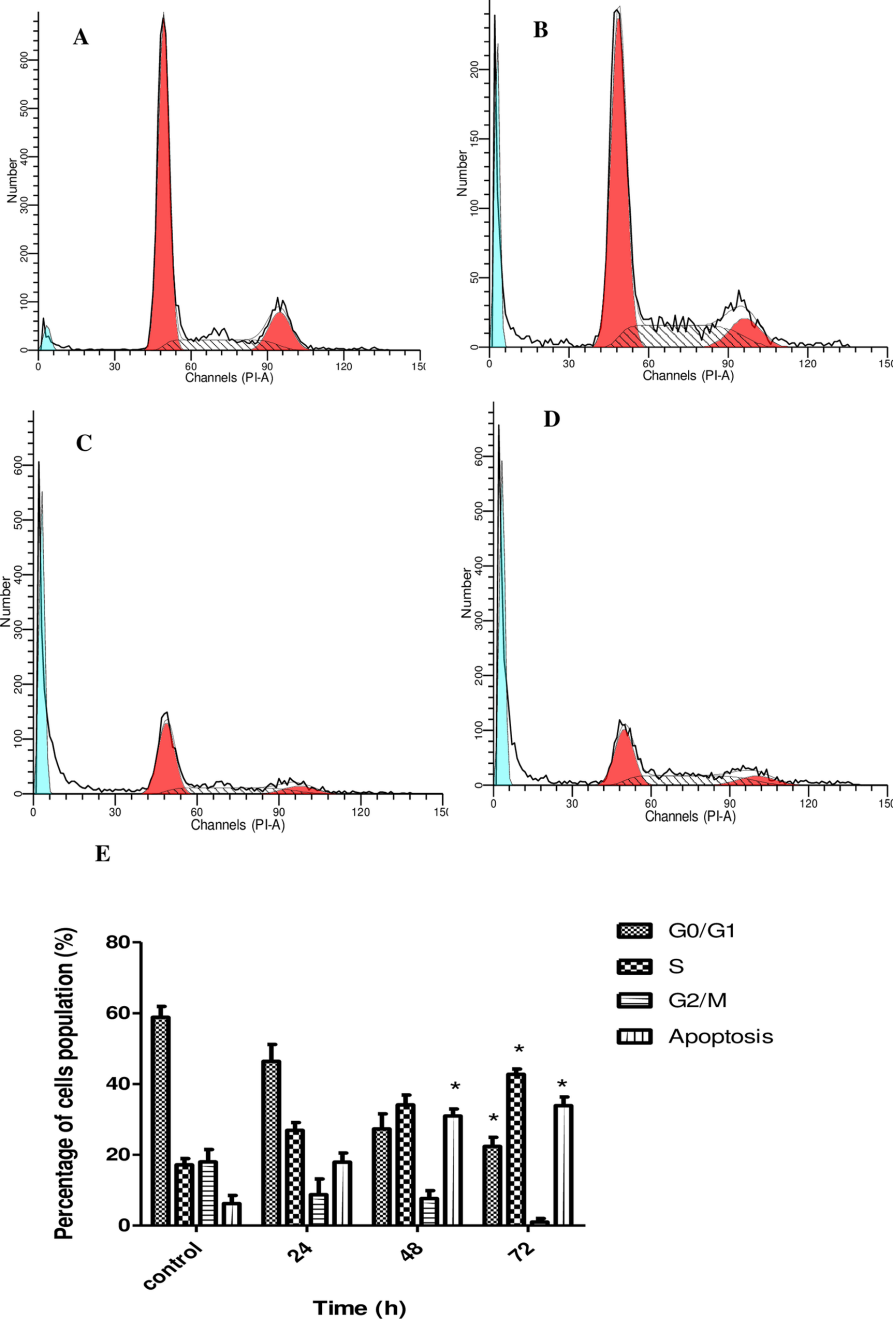
Flow cytometry analysis of cell cycle phase distribution of SKOV-3 cells treated with artonin E (8 μg/mL) in a time-dependent manner. [A] Untreated cells, [B] cells after 24 h treatment, [C] cells after 48 h treatment, and [D] cells after 72 h treatment. [E] Graphical analysis represented cell cycle arrest in SKOV-3 cells. Results are represented as mean±SD of three replicates. * indicates significant difference from the control of each phase (*p<*0.05).

Flow cytometry analysis was employed to investigate whether the antiproliferative effect of artonin E on SKOV-3 cells is partially correlated with cell cycle arrest. Results confirm that artonin E induced a depletion of SKOV-3 cells in the S phase in a time-dependent manner. The accumulation of cells arrested at the S phase was significantly (*p<0*.*05*) increased at 72 h after treatment with a concurrent decrease in the proportion of cells in the G0/G1 phase. Results also indicate that the proportion of cells in the apoptosis stage increased significantly (*p<0*.*05*) (Fig 7) in a time-dependent manner.

### Artonin E induces ROS formation

**Fig 8:**
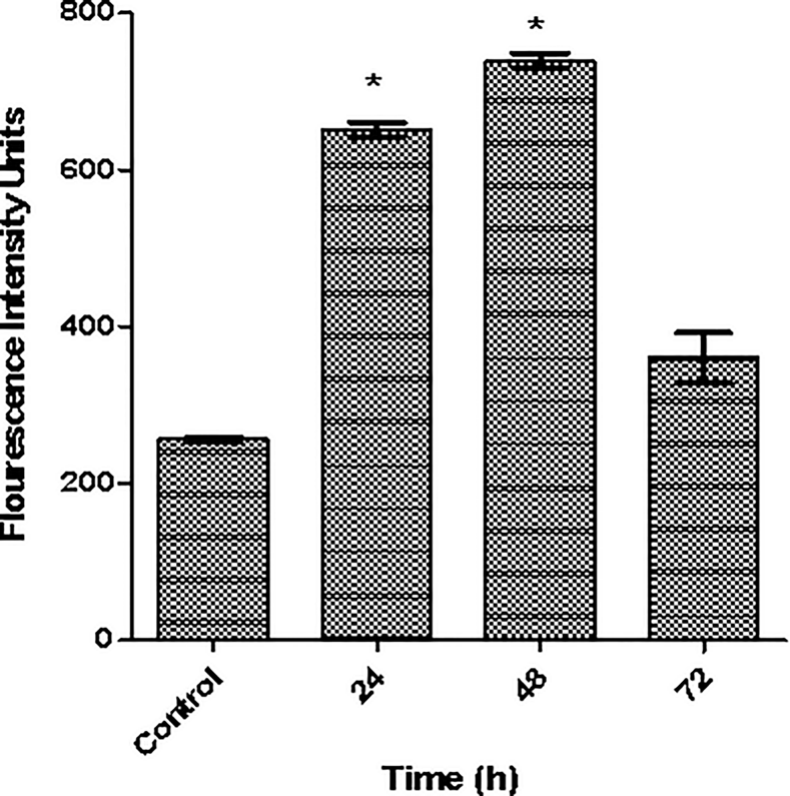
Effects of Artonin E on ROS generation in SKOV-3 cells. Cells were treated with 8 μg/mL of artonin E for 24, 48, and 72 h. Values are expressed as mean±SD from three independent experiments. Statistical significance was expressed as *p<0.*05*.

Reactive oxygen species (ROS) are products of normal metabolism during the reduction of oxygen to water. Depending on their concentrations, ROS can be beneficial or harmful to cells and tissues. Excessive ROS production as a result from oxidative stress leads to a various biochemical and physiological impairments and promotes cell death. There are several anticancer drugs and natural compounds such as paclitaxel, cisplatin, curcumin and quercetin previously reported as ROS up-regulating agents[30]. Therefore, it is an upsurge of interest to evaluate the ROS formation in SKOV-3 cells treated with artonin E. A time-dependent generation of intracellular ROS was markedly increased as shown in Fig 8. At 24 and 48 h after treatment, the treated cells displayed a twofold (*p<0*.*05*) increase in the formation of ROS compared with untreated cells. However, the ROS induction was slightly decreased at 72 h after treatment. A slight reduction of ROS at 72 h showed that the cells entered to the late phase of apoptosis, which indicating that the mitochondria in the cells were partially dysfunction and some of cells were already death[31].The present finding suggests that artonin E partially induces apoptosis through the production of intracellular ROS.

### Artonin E induces mitochondrial membrane potential disruption and release of *cytochrome c*

**Fig 9:**
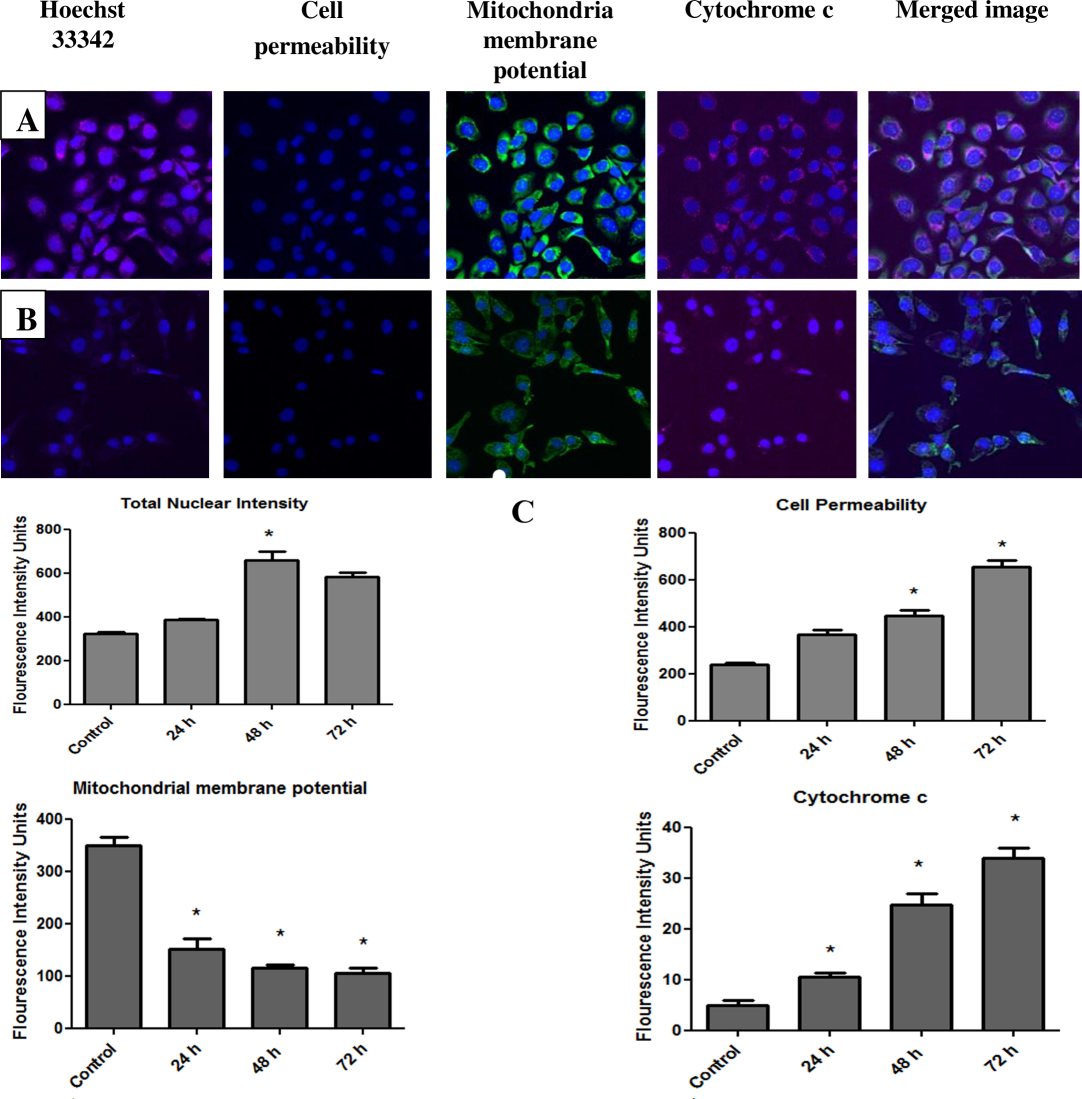
Representative image of SKOV-3 cells in a 96-well black plate and exposed to either medium alone or 8 μg/mL artonin E for 24, 48, and 72 h. The treated cells showed reduction in cell number, increase in total nuclear intensity, increase in cell membrane permeability, loss of MMP, and increase in cytochrome c release. Images were observed at magnification 20×. (A) Untreated SKOV-3 cells and (B) SKOV-3 cells treated with 8 μg/mL of artonin E. (C) Time-dependent quantitative analysis of SKOV-3 cells treated with artonin E for different apoptosis parameters. Average intensities were observed simultaneously in SKOV-3 cells for total nuclear intensity, cell permeability, MMP, and cytochrome *c* release. All data were expressed as means±SD. *p<0.05 indicates significant difference from control.

Multi-parameter cytotoxicity 3 assay was performed to verify the presence of apoptosis in morphological and cellular apoptosis as shown in AO–PI and Annexin V assays. In addition, it is well documented that accumulation of ROS inside the cells could result in the disruption of plasma membrane and mitochondrial damage. In this assay, we then further investigate the effect of artonin E on cell permeability, mitochondrial membrane potential (MMP) changes and cytochrome *c* localization and release from mitochondria. Hoechst and MMP fluorescence probe were used to evaluate the function of nucleus and mitochondria. As shown in Fig 9, the untreated cells showed intact nuclei, whereas the treated cells exhibited clear nuclear condensation, as seen in hoechst 33342 staining. The alterations in nuclear intensity, which are closely associated with apoptotic chromatin changes, such as membrane blebbing, fragmentation, and condensation, are quantified in Fig 9C. Time-dependent significant *(p<0*.*05)* reduction of green fluorescence intensity of MMP was associated with the marked increased in cells permeability (Fig 9C). These changes were also associated with the collapse of MMP (Fig 9). In addition, an increase in cytochrome c fluorescence intensities was also observed after prolonged treatment periods.

**Fig 10:**
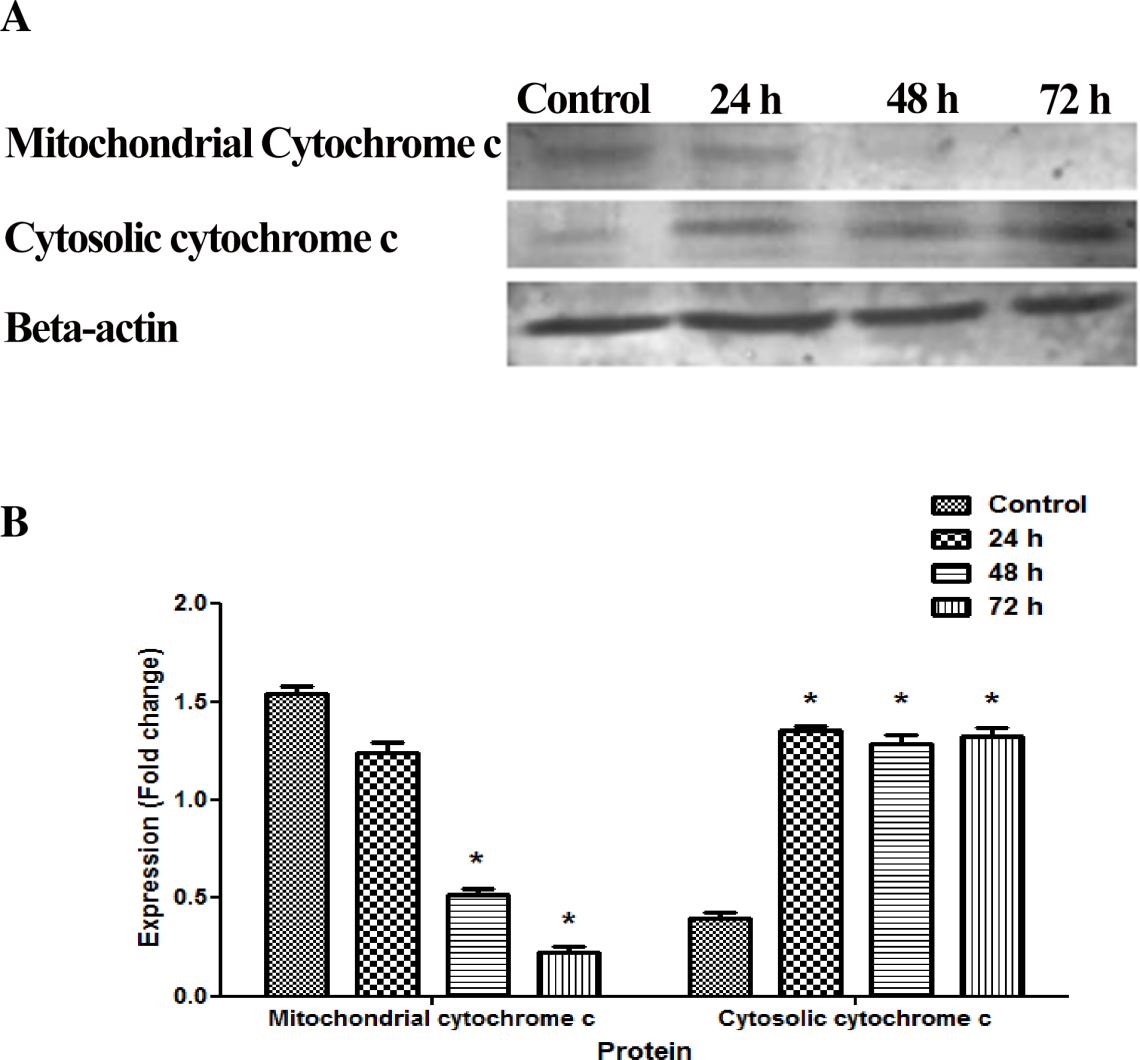
(A) Effect of artonin E (8 μg/mL) on the mitochondrial and cytosolic cytochrome c fractions after 24, 48, and 72 h. β-Actin was used as loading control. (B) Quantitative analysis of SKOV-3 cells treated with artonin E. All data were expressed as mean±SD. Statistical significance was expressed as *p<0.05.

Next, to investigate the translocation of cytochrome c from mitochondria into the cytososl, we then analyzed the expression level cytochrome c in the cytosolic and mitochondrial fractions by using Western blot. As shown in Fig 10A, artonin E significantly *(p<0*.*05)* decreased the expression level of cytochrome c in mitochondrial fraction c in a time-dependent fashion. Consistent with this, an increase in the expression level of cytochrome c in cytosolic fraction was also observed, thus suggesting that artonin E triggered the release of cytochrome c from mitochondrial into cytosol. Taken together, the present results exhibited time-dependent significant *(p<0*.*05)*increased upon artonin E treatment in total nuclear intensity, increased in cell permeability, collapsed of MMP and elevated translocation of cytochrome *c* in the cytosol when compared to the control. In addition, the disruption of MMP in the apoptotic cells has been linked with rapid generation of ROS; hence, these findings propose that artonin E induced apoptosis through mitochondrial mediated intrinsic pathway.

### Artonin E induces caspases activation

**Fig 11:**
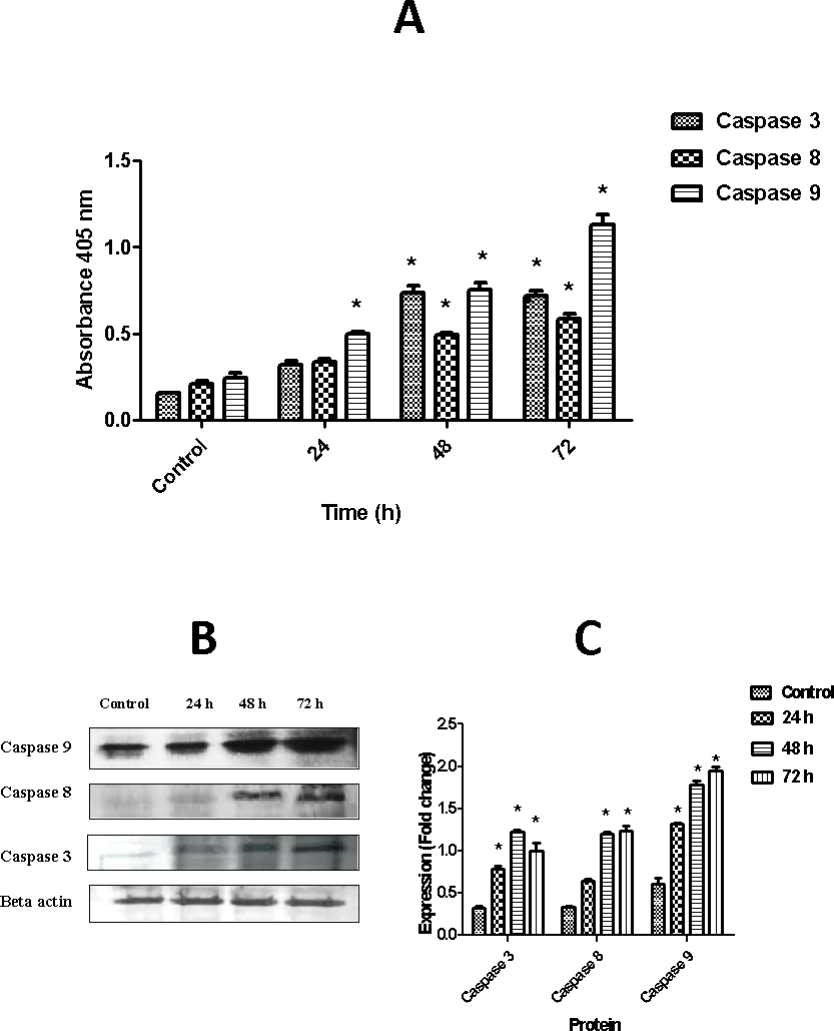
Effect of artonin E on caspases activation. (A) Relative expression levels of caspases- 3, -8, and -9 in SKOV-3 cells treated with 8 μg/mL of artonin E for 24, 48, and 72 h. Results are represented as mean±SD of three replicates. *p<0.05 indicates significant difference from control. (B) Time-dependent of protein expression levels of caspase-3, -8 and -9 in SKOV-3 cells treated with 8 μg/mL of artonin E. Artonin E induced the up-regulation of caspase-9, -3 and -8. (C) The quantitative analysis is expressed as a ratio to the expression of β-actin. The data are represented as mean±SD of three replicates. *p<0.05 indicates significant difference from control.

Caspases are crucial effector molecules in apoptosis[32]. To date, most of anticancer strategies used in clinic have been associated with the activation of intrinsic and/ or extrinsic caspases pathway[33]. In this study, we investigated time-dependent activation of caspase -3, -8 and -9 in SKOV-3 cells treated with artonin E. Time-dependent increases in the activation of caspases-3, -8, and -9 were detected, as shown in Fig 11A. The stimulation of caspase-9 appeared as early as 24 h after treatment and increased significantly (*p<0*.*05*)after prolonged exposure time. Meanwhile, the activation of caspase-3 and -8 were significantly (*p<0*.*05*)elevated at 48 and 72 h treatment. These results propose the involvement of caspase cascades in artonin E mediated apoptosis in SKOV-3 cells. The western blot analysis of caspases also demonstrated that artonin E significantly (*p<0*.*05*) up-regulated the protein expression level of caspase-9 and -3 as early as 24 h after treatment and increase after prolonged incubation period (Fig 11B & 11C). In contrast, the protein expression level of caspase-8 was up-regulated after 48 and 72 h. High expression level of caspase-9 and -3 as shown in calorimetric and western blot assays suggest that artonin E induced apoptosis predominantly through mitochondrial mediated intrinsic pathway.

### Artonin E induces DNA fragmentation

**Fig 12:**
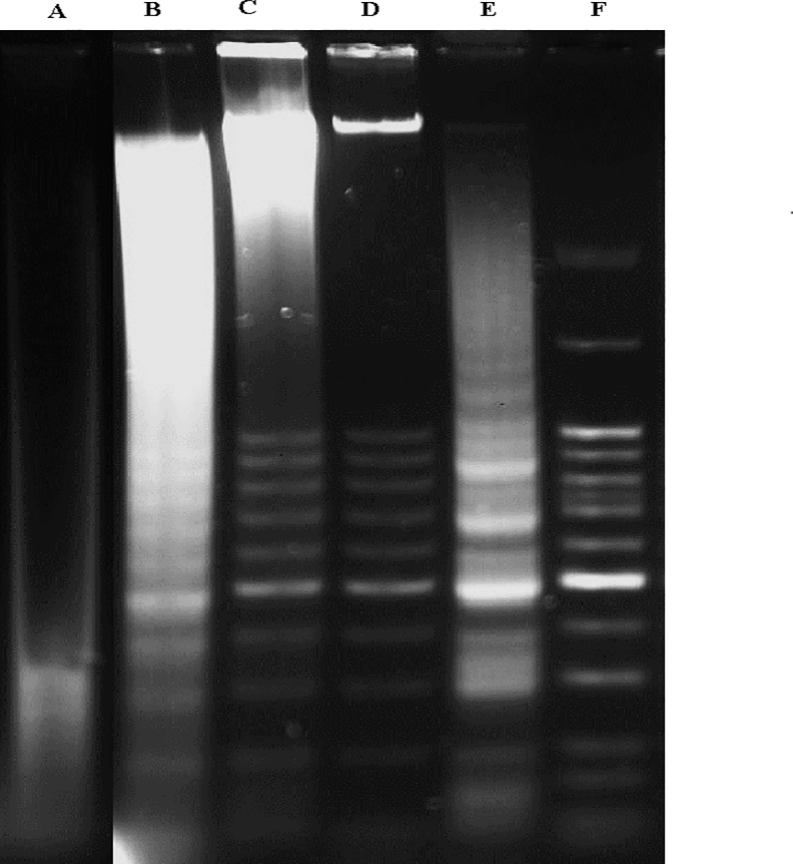
Time-dependent electrophoresis gel separation of DNA isolated from SKOV-3 cells. The cells were treated with artonin E(8 μg/mL). Lane A: untreated cells; lane B: SKOV-3 cells treated with artonin E for 24 h; lane C: cells after 48 h treatment; lane D: cells after 72 h treatment; lane E: positive control (HL-60 cells treated with actinomycin D); and lane F: DNA marker (50 base pairs).

DNA fragmentation was distinctly observed in SKOV-3 treated cells on 1.5% agarose gel electrophoresis. The results also demonstrate a time-dependent increase in the DNA ladder pattern after prolonged exposure time. As shown in Fig 12, no ladder appeared in the untreated cells, whereas in the positive control, the ladder was visible. This result confirms that artonin E activated apoptosis in SKOV-3 cells with chromosomal DNA cleaving into oligonucleasomal size fragments as an essential part of apoptosis induction.

### Artonin E upregulates Bax and suppresses the expression of Bcl-2, HSP70, and survivin

**Fig 13:**
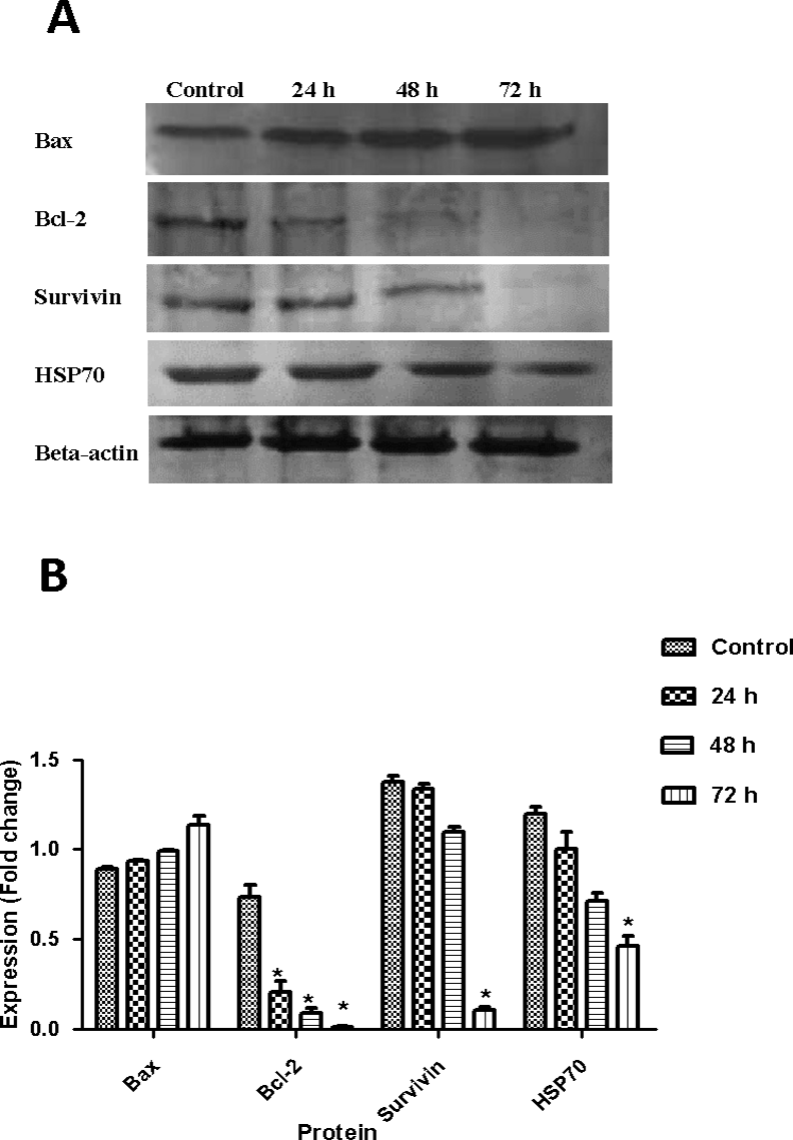
(Top) Effect of artonin E (8 μg/mL) on the apoptosis protein expression after 24, 48, and 72 h. β-Actin was used as loading control. (Bottom) Quantitative analysis of SKOV-3 cells treated with artonin E. All data were expressed as mean±SD. Statistical significance was expressed as *p<0.05.

Western blot analysis was used to examine the expression levels of pro- and anti-apoptotic proteins Bax, Bcl-2, HSP70, and survivin in SKOV-3 cells treated with artonin E. The results in Fig 13 exhibited that artonin E reduced the expression levels of anti-apoptotic proteins Bcl-2, HSP70, and survivin in a time-dependent manner. At 24 h post treatment, the Bcl-2 level was significantly lower and had almost diminished after 48 h and 72 h. The levels of HSP70 and survivin expression were reduced significantly 72 h after treatment. The time-dependent effect was also observed in the expression of pro-apoptotic protein Bax in SKOV-3 cells exposed to artonin E.

## Discussion

In Malaysia, a wide variety of herbal remedies for a number of diseases has been traditionally passed from generation to generation as part of its living heritage. Many of the herbal remedies used by local people, such as *Curcuma longa* and *Centellia asiatica*, have been scientifically proven to exhibit ethno-medicinal and healing properties [34]. *A*.*elasticus* is one of the medicinal plants used in Malaysia and other Southeast Asian countries to treat ringworm, diarrhea, malaria, tuberculosis, inflammation, and tinea versicolor [35, 36]. The present study demonstrates that artonin E isolated from the stem bark of *A*. *elasticus* inhibits the proliferation of human ovarian cancer cell lines (SKOV-3) via apoptosis. Although artonin E has been reported to restrain several cancer cells, such as lung, breast, and leukemia cell lines, no information concerning the apoptotic activities and related mechanism involved of artonin E in human ovarian cancer cells has been reported. Thus, this study is the first to demonstrate the apoptotic effect of artonin E on SKOV-3 cells via mitochondria-dependent activation of the caspase cascade, increased ROS level, inhibition of colony formation, and S phase cell cycle arrest.

MTT assay revealed that artonin E inhibited the proliferation of SKOV-3 cells. Results also indicate that normal cell lines tested were more resistant to artonin E-mediated cytotoxicity activity than SKOV-3 cells. A clonogenic assay was employed to evaluate cell survival after drug treatment. Clonogenic cell survival assay is one of the methods used to examine cell reproductive death after the cells have been exposed to a certain drug or to ionizing radiation [37]. In this assay, the unaffected cells proliferated indefinitely until they formed colonies, whereas the affected cells stopped dividing and eventually died from loss of their reproductive integrity [38]. As shown in MTT and colony formation assays, the capability of artonin E to inhibit SKOV-3 cells, with high resistance by normal cells, suggests that this compound has a selective effect toward cancerous and normal cells. According to Blagosklonny (2005), an ideal anticancer drug must be cytotoxic to cancer cells and selective toward normal cells[39].

A morphological study was used to evaluate the mode of cell death induced by artonin E. Normal inverted microscopy showed that cell number decreased after prolonged exposure to artonin E. The early and late phases of apoptosis in cells treated with artonin E were morphologically identified with AO–PI double staining. A significant *(p<0*.*05)* time-dependent increase in apoptotic population was observed by Annexin V flow cytometry for both phases. An underlying mechanism of artonin E induced apoptosis in SKOV-3 cells seems to be closely associated with its effect on the cell cycle. The cell cycle is a tightly regulated process, and a defect in this process leads to tumorigenesis [40].Therefore, understanding the cell cycle process in cancer is critical in improving current cancer treatment. In this study, artonin E was shown to induce S phase cell cycle arrest in SKOV-3 cells. The S phase is a critical phase in the cell cycle, in which DNA synthesis occurs [41]; hence, a compound or drug that exhibits S phase cell arrest also induces apoptosis [42, 43]. Thus, the modes of artonin E-induced cell death in SKOV-3 cells were confirmed to be apoptosis and cell cycle arrest at S phase.

ROS and mitochondria have recently attracted considerable scientific attention because of their important role in apoptosis and cancer [44, 45]. The apoptotic effect of artonin E on SKOV-3 cells is closely related to a notably increased level of intracellular ROS. Elevated levels of ROS contribute to stress sensing, which triggers apoptosis[46]. Oxidation of the mitochondrial pores because of rapid generation of ROS may result in disruption of the MMP, which is an early indication of mitochondrial changes [47, 48]. ROS is an intrinsic death stimulus that directly or indirectly activates the mitochondrial pathway by activation of cytochrome *c* and formation of apoptosome [49].

SKOV-3 cells treated with artonin E had significantly increased total nuclear intensity, cell permeability, and cytochrome *c* release from mitochondria into cytosol, as shown by multi-parametric apoptosis analysis and Western blot. The changes in MMP were significantly observed in treated cells. The mitochondria assimilate various signals, including endogenous and exogenous factors, which consequently cause initiation of MMP. The opening of the mitochondrial permeability transition pore has been linked to enhanced permeability and loss of MMP. Hence, the role of mitochondria in SKOV-3 cell apoptosis was investigated by detecting the changes in MMP because the release of mitochondrial apoptotic factors, such as cytochrome *c*, is influenced by the opening of the permeability transition pore and its consequences [50]. The current results are in good agreement with those in the literature, suggesting that artonin E may affect the mitochondria, leading to apoptosis.

Caspases are a family of endoproteases that play a critical role in maintaining tissue homeostasis through regulation of cell death and inflammation [32]. An increasing evidence suggests that caspases can be exploited in reinstating apoptosis signaling toward selective targeting of malignant cells [51]. Artonin E significantly *(p<0*.*05)* induced the activation of caspases-3, -8, and -9 in SKOV-3 cells. The increased expression levels of caspases-9 and -3 in this study suggest that artonin E induced apoptosis predominantly through mitochondria-mediated intrinsic pathway. The intrinsic pathway is initiated by the mitochondrial release of cytochrome *c*, thus recruiting caspase-9, whereas the extrinsic pathway activates caspase-8 [49]. The activation of caspase-9 and the significant release of cytochrome *c* detected earlier shows the substantial role of the mitochondria in artonin E-mediated apoptosis.

The endonuclease cleavage product was then investigated using DNA laddering gel electrophoresis because the caspase cascade leads to apoptosis via fragmentation of the DNA. DNA fragmentation is a biochemical endpoint of apoptosis after the cell has committed suicide [27]. Based on the current results, a time-dependent increasing in DNA fragmentation was observed, thus suggesting that the mode of cell death induced by artonin E is confirmed to be apoptosis.

Western blot was employed to investigate the role of Bcl-2 and Bax because of the apparent role of mitochondria in artonin E-mediated apoptosis. The Bcl-2 family is the central regulator of apoptosis, which comprises both pro- and anti-apoptotic activities through the regulation of the mitochondrial pathway [52]. Members of this family such as Bcl-2/Bax are in intricate associations with one another in indicating the survival or death of a cell by controlling mitochondrial membrane permeabilization [53]. The repossession of Bcl-2 with Bax helps in preventing apoptosis, which was exhibited in SKOV-3 cells treated with artonin E by downregulating Bcl-2 and upregulating Bax protein.

Survival pathways also play a crucial part in determining the fate of cells going through apoptosis. Survivin is a cell survival promoter that belongs to the anti-apoptotic protein family, which regulates apoptosis and cell division. This protein is expressed during embryonic and fetal development. Survivin is highly expressed in ovarian cancers, but not in normal ovarian tissues [54]. High expression of survivin is found in most solid tumors and is closely associated with poor diagnosis, increased risk of recurrence, development of drug resistance, lymph node invasion, and metastasis [55]. Hence, decreased expression of survivin in SKOV-3 cells treated with artonin E led to an increase in apoptosis and decrease in cancer cell growth.

Exposure of SKOV-3 cells to artonin E significantly *(p<0*.*05)* reduced the expression of HSP70 protein. HSPs are apoptosis inhibitors that highly conserve proteins induced by a variety of stresses [56, 57]. Recent evidence suggests that HSP70 prevents Bax translocation from the cytosol to the mitochondria, which is correlated with cisplatin-resistance in ovarian cancer [56]. A high expression of HSP70 inhibits stress-induced JNK signaling pathway, cytochrome *c* release, apoptosome formation, caspase activation, and nuclear uptake of AIF [58]. As such, inhibition of HSP70 by artonin E could be important in inducing apoptosis in SKOV-3 cells.

## Conclusion

In conclusion, this study recommends that artonin E be considered a potent compound that affects apoptosis in SKOV-3 ovarian cancer cells. Artonin E-induced apoptosis is accomplished through multiple signaling pathways, such as the intrinsic caspase pathway, Bcl-2/Bax, survivin, HSP70 signaling pathways, and cell cycle arrest. These findings provide basis for determining the precise mechanism of artonin E-induced apoptosis in a pre-clinical animal model.
